# Regular Humoral and Cellular Immune Responses in Individuals with Chronic Myeloid Leukemia Who Received a Full Vaccination Schedule against COVID-19

**DOI:** 10.3390/cancers15205066

**Published:** 2023-10-20

**Authors:** Sara Rodríguez-Mora, Magdalena Corona, Miriam Solera Sainero, Elena Mateos, Montserrat Torres, Clara Sánchez-Menéndez, Guiomar Casado-Fernández, Javier García-Pérez, Mayte Pérez-Olmeda, María Aranzazu Murciano-Antón, Javier López-Jiménez, Mayte Coiras, Valentín García-Gutiérrez

**Affiliations:** 1 Immunopathology Unit, National Center of Microbiology, Instituto de Salud Carlos III, 28220 Madrid, Spain; 2 Biomedical Research Center Network in Infectious Diseases (CIBERINFEC), Instituto de Salud Carlos III, 28029 Madrid, Spain; 3 Hematology and Hemotherapy Service, Instituto Ramón y Cajal de Investigación Sanitaria (IRYCIS), Hospital Universitario Ramón y Cajal, 28034 Madrid, Spain; 4 Faculty of Sciences, Universidad de Alcalá, 28801 Madrid, Spain; 5 AIDS Immunopathology Unit, National Center of Microbiology, Instituto de Salud Carlos III, 28220 Madrid, Spain; 6 Serology Service, Instituto de Salud Carlos III, 28029 Madrid, Spain; 7 Family Medicine, Centro de Salud Doctor Pedro Laín Entralgo, 28924 Alcorcón, Spain

**Keywords:** humoral immune response, cellular immune response, chronic myeloid 24 leukemia, COVID-19 vaccination, SARS-CoV-2, oncohematological disease

## Abstract

**Simple Summary:**

Individuals with chronic myeloid leukemia (CML) are different from other individuals with oncohematological disease (OHD) because they receive treatment with drugs that may modulate the activity of cells from the immune system, causing an increase in their activity against the cancerous cells. This activity may also be effective against cells infected with virus. Although people with OHD usually have a reduced response to viral infections, individuals with CML present low risk of infection. Therefore, we hypothesized that people with CML may develop a better response after vaccination against COVID-19 than other individuals with OHD. We confirmed that there was no difference between people with CML and healthy donors in the formation of antibodies against SARS-CoV-2 with capacity to neutralize the virus after receiving vaccination. Similar results were also observed between both groups in the cellular immunity. In conclusion, individuals with CML developed immunity that was comparable to healthy donors after COVID-19 vaccination, although it was better in people with CML who was still on treatment against their cancer disease than in those who had discontinued treatment due to great improvement in the CML. And although the vaccination did not impede completely infections with SARS-CoV-2 in individuals with CML, it prevented the development of severe or critical illness.

**Abstract:**

Individuals with chronic myeloid leukemia (CML) constitute a unique group within individuals with oncohematological disease (OHD). They receive treatment with tyrosine kinase inhibitors (TKIs) that present immunomodulatory properties, and they may eventually be candidates for treatment discontinuation under certain conditions despite the chronic nature of the disease. In addition, these individuals present a lower risk of infection than other immunocompromised patients. For this study, we recruited a cohort of 29 individuals with CML in deep molecular response who were on treatment with TKIs (n = 23) or were on treatment-free remission (TFR) (n = 6), and compared both humoral and cellular immune responses with 20 healthy donors after receiving the complete vaccination schedule against SARS-CoV-2. All participants were followed up for 17 months to record the development of COVID-19 due to breakthrough infections. All CML individuals developed an increased humoral response, with similar seroconversion rates and neutralizing titers to healthy donors, despite the presence of high levels of immature B cells. On the whole, the cellular immune response was also comparable to that of healthy donors, although the antibody dependent cytotoxic activity (ADCC) was significantly reduced. Similar rates of mild breakthrough infections were observed between groups, although the proportion was higher in the CML individuals on TFR, most likely due to the immunomodulatory effect of these drugs. In conclusion, as with the healthy donors, the vaccination did not impede breakthrough infections completely in individuals with CML, although it prevented the development of severe or critical illness in this special population of individuals with OHD.

## 1. Introduction

Coronavirus disease 19 (COVID-19), caused by the emergent virus SARS-CoV-2, was declared a pandemic by the World Health Organization (WHO) in March 2020 [1]. Individuals with oncohematological diseases (OHDs) were reported to be a high-risk group for COVID-19 infection, with a mortality rate of over 30% [2,3,4]. The approval of SARS-CoV-2 vaccines changed the landscape of the pandemic, although their efficacy was impaired in these highly immunosuppressed individuals. Consequently, the analysis of the efficacy of the immune response developed in individuals with OHDs after receiving the full vaccination schedule against SARS-CoV-2 is a priority. 

Chronic myeloid leukemia (CML) is a unique disease within the spectrum of oncohematological neoplasms. This disease is usually treated with a family of compounds named tyrosine kinase inhibitors (TKIs) that are characterized by adequate immunomodulatory properties [5]. TKIs target and inhibit the constitutively activated tyrosine kinase pathways of BCR::ABL1 oncogene, which is the trigger of CML. 

CML individuals are usually categorized as immunosuppressed individuals because of their oncohematological neoplasm, which entails an accumulation of immature myeloid cells that suppress the innate and adaptive immune system, the myeloid-derived suppressor cells (MDSCs), the regulatory T-cells and the aberrant expression of immune checkpoint signaling pathways, leading to an inhibition of the host-antitumor immunity [6]. However, this impairment is usually completely reversed during TKI therapy. In fact, some TKIs, such as imatinib and nilotinib, are quite selective of BCR::ABL1, whereas other TKIs, such as bosutinib, dasatinib, and ponatinib present off-target effects over other tyrosine kinases that may stimulate the immune response [7]. It has been described that TKIs exhibit an action against MDSCs and regulatory T-cell populations, conferring immune system reactivation and restoring effector-mediated immune surveillance [6]. Furthermore, increased levels of functional large granular lymphocytes (LGLs) with natural killer (NK) cell profiles are developed during TKI treatment [8,9,10,11], and their presence may be related to a sustained treatment free-response (TFR) after discontinuation due to the antitumoral effect of these cells [12]. This immunomodulatory capacity of TKIs is also applied to other cells of the immune system, such as B cells and T cells. However, in this case, TKI may present a detrimental effect to the immune response due to a B-cell impairment with reduced memory B-cell frequency as a consequence of the suppressive effect of some TKIs on the Bruton tyrosine kinase. This may decrease the humoral response against vaccines [13], and produce a potent cytostatic effect on T lymphocytes [14,15].

Therefore, the immune system activity of these individuals and their response to vaccines are expected to be completely different from that of other OHDs. Moreover, this response could be different in individuals with CML who are still on treatment with TKIs in comparison to those who discontinued therapy due to sustained deep molecular response (DMR, BCR::ABL levels ≤ 0.01%); it is uncertain if the immunomodulatory effect of TKIs persists after discontinuation as well as its impact on the response to the vaccine and the severity or incidence of breakthrough infections.

Accordingly, in this study, we evaluated the humoral and cellular immunity developed in individuals with CML on treatment with TKI or TFR in response to the complete vaccination schedule against SARS-CoV-2. We aimed to correlate these results with the clinical outcomes of breakthrough infections during vaccination. This knowledge is essential to improve the clinical management of this group of individuals with OHD, especially since new viral variants of SARS-CoV-2 have cast doubt on the efficacy of the long-term protection acquired through previous infection or complete vaccination [16,17].

## 2. Materials and Methods

### 2.1. Study Populations

This was an observational, prospective, longitudinal, single-center study. Adult individuals diagnosed with CML with a DMR status disease (n = 29) were recruited at the Hospital Universitario Ramon y Cajal (Madrid, Spain) before receiving the first dose of the vaccination schedule against COVID-19 in January 2021. Discontinuation of TKIs was performed at their medical team’s discretion, following the recommendations of the European Leukemia Net (ELN) [12]. ELN definitions also state CML individual’s molecular response and TFR.

Healthy individuals (n = 20) matched through age and gender who received a similar vaccination schedule were recruited as controls at the Primary Healthcare Center Doctor Pedro Laín Entralgo (Madrid, Spain). All individuals received two doses of one of the FDA/EMA approved vaccines against COVID-19 (Comirnaty, BioNTech/Pfizer, Spikevax, Moderna, Vaxzevria, AstraZeneca) through the Spanish Vaccination Program. None of the participants had a previous history of infection with SARS-CoV-2. A basal serology test against SARS-CoV-2 was performed in the first blood sample in order to identify and exclude those individuals with a history of asymptomatic COVID-19.

Blood samples were obtained from all participants before vaccination and one month after receiving the second dose. After collection, the blood samples were immediately processed at the Instituto de Salud Carlos III (Madrid, Spain).

### 2.2. Ethical Statement

This study was approved by the Ethics Committee of the Hospital Universitario Ramon y Cajal (favorable report number 053-21) and the Primary Care Assistance Management of Comunidad de Madrid (Madrid, Spain) (favorable report number 20210008; CCI 20210017). All individuals gave informed written consent to participate in the study in accordance with the Helsinki declaration. Confidentiality and anonymity were protected by the current Spanish and European Data Protection Acts.

### 2.3. Breakthrough Infections

Participants were followed up for 17 months after receiving the second dose of vaccine through electronic medical history and periodic phone calls to register breakthrough infections after receiving the vaccination against SARS-CoV-2. We registered adjuvant treatment administered for the infection, hospital admission, incidence of pneumoniae, oxygenation impairment needing respiratory assistance, and ICU admission. COVID-19 infection severity was categorized based on the WHO disease severity classification of mild, moderate, severe, or critical [18].

### 2.4. Blood Sample Processing and Cell Lines

Peripheral blood lymphocytes (PBMCs) and plasmas were isolated and cryopreserved after processing blood samples via centrifugation through Ficoll-Hypaque gradient (Pharmacia Corporation, North Peapack, NJ, USA). Raji cell line (ATCC CCL-86) was provided by the existing collection of the Instituto de Salud Carlos III (Madrid, Spain). Vero E6 cell line (ECACC 85020206) was kindly provided by Dr. Antonio Alcami (CBM Severo Ochoa, Madrid, Spain). Vero E6 and HEK-293T (National Institute for Biological Standards and Control (NIBSC, Potters Bar, UK) cells were cultured in DMEM supplemented with 10% FCS, 2 mM L-glutamine, and 100 units/ml penicillin/streptomycin (Lonza, Basel, Switzerland).

### 2.5. Phenotyping of B Lymphocytes

B-cell subpopulations (CD3^−^CD19^+^) were differentiated via flow cytometry after staining the surface markers of CD10, CD27, CD20, and CD21 as follows: immature or transitional cells (CD10^+^CD27^−^); naïve B cells (CD10^−^CD27^−^CD21^high^); tissue-like memory cells (CD10^−^CD27^−^CD21^low^); resting memory cells (CD10^−^CD27^+^CD21^high^); activated memory cells (CD10^−^CD27^+^CD21^low^); and plasmablasts (CD27^++^CD20^−^CD21^low^). Antibodies CD3-PE, CD10-BV421, CD19-BV711, CD20-AlexaFluor700, CD21-FITC, and CD27-PercPCy5.5 were purchased from BD Biosciences (San Jose, CA, USA). Data acquisition was performed in a BD LSRFortessa X-20 flow cytometer with FACS Diva software v7.0 (BD Biosciences, San Jose, CA, USA). FlowJo software v10 (Tree Star Inc., Ashland, OR, USA) was used for data analysis.

### 2.6. SARS-CoV-2 Serology

IgG antibodies against the S1 domain of the SARS-CoV-2 spike protein were measured in plasma via Euroimmun Anti-SARS-CoV-2 ELISA Assay (Euroimmun, Lübeck, Germany). The results obtained corresponded to a semi-quantitative measure calculated as the ratio of the optical density (OD) of each sample over the OD of a calibrator included in the assay. Results were considered positive with IgG titers of >1.1; values between 0.8 and 1.1 were considered undetermined and were not used in the analysis and values of <0.8 were considered negative.

### 2.7. Neutralization Assays

Pseudotyped SARS-CoV-2 virus pNL4-3Δenv_SARS-CoV-2-SΔ19(G614)_Ren was used to assess the neutralizing capacity of IgGs detected in the plasma of all participants [19]. This single-cycle virus expressed the HIV-1 genome and the renilla luciferase gene as a reporter. In order to evaluate the neutralization activity, pseudotyped SARS-CoV-2 (10 ng p24 Gag per well) was pre-incubated for 1 h at 37 °C with 4-fold serial dilutions (1/32 161 to 1/8192) of decomplemented IgG-positive plasma and then co-cultured for 48 h with a monolayer of Vero E6 cells. Viral infectivity was assessed in lysed Vero E6 cells via Renilla Luciferase Assay kit (Promega, Madison, WI, USA) and a luminometer Centro XS3 LB 960 with MikroWin 2010 software v5.24 (Berthold Technologies, Baden-Württemberg, Germany). Titers of neutralizing antibodies were represented as 50% inhibitory dose (ID50) using non-linear regression in GraphPad Prism Software v9.4.0 (GraphPad, Inc., San Diego, CA, USA).

### 2.8. Antibody-Dependent Cellular Cytotoxicity Assay

Raji cell line, which was provided by the existing collection of the Instituto de Salud Carlos III (Madrid, Spain), was used as a target to determine the antibody-dependent cellular cytotoxicity (ADCC) capacity of PBMCs isolated from CML individuals and healthy donors, as described previously [20]. Briefly, Raji cells were labeled with PKH67 green fluorescent cell linker (Merck KGaA, Darmstadt, Germany) and then coated with rituximab (50 μg/mL) (Selleckhem, Houston, TX, USA) for 4 h. Labeled rituximab-coated Raji cells were then co-cultured for 18 h with PBMCs (1:2 ratio). Early apoptosis of Raji cells was evaluated via staining with Annexin V conjugated with phycoerythrin (PE) (Immunostep, Salamanca, Spain). Data acquisition was performed with a BD LSRFortessa X-20 flow cytometer and data analyses were performed with FlowJo software v10 (Tree Star Inc.).

### 2.9. Direct Cellular Cytotoxicity Assay

For the analysis of direct cellular cytotoxicity (DCC), Vero E6 cells were infected with equal amounts (100 ng p24 Gag/well) of the most important variants of SARS-CoV-2 within clade 19B that were circulating in Spain at the time of the study: D614 and G614 [21]. After 48 h of incubation, Vero cells were co-cultured for 1 h with PBMCs from CML individuals and healthy donors (ratio 1:1). After detaching the Vero monolayer with the trypsin-EDTA solution (Sigma Aldrich-Merck, Darmstadt, Germany), caspase-3 activity was measured via luminescence using a Caspase-Glo 3/7 Assay system (Promega). Cytotoxic cell populations such as NK, NKT-like, and Tγδ cells were analyzed in the supernatants using specific conjugated antibodies: CD3-PE, CD56-BV605, CD16-PercP, CD8-APC H7, CD107a-PE-Cy7, and TCRγδ-FITC (BD Biosciences). Data acquisition was performed in a BD LSRFortessa X-20 flow cytometer and FACS Diva software v7.0 (BD Biosciences). FlowJo software v10 (Tree Star Inc.) was used for data analysis.

### 2.10. Statistical Analysis

Statistical analysis was performed using GraphPad Prism 9.0 (GraphPad Software Inc., San Diego, CA, USA). The Kolmogorov—Smirnov test was used to evaluate the normal distribution of variables. The quantitative variables were represented as mean and standard deviation of the mean (SEM). Significance in the comparison between groups was analyzed using a *t*-test and a Wilcoxon signed-rank test. The unpaired groups were compared using a Kruskal—Wallis one-way, ordinary one-way ANOVA analysis and a Mann—Whitney U test of variance by ranks was applied to compare between pre- and post-vaccination samples. *p* values (*p*) < 0.05 were considered statistically significant in all comparisons.

## 3. Results

### 3.1. Participants’ Characteristics

We recruited 29 individuals diagnosed with CML for a median of 10 years (IQR 4-13). The median age was 65 years old (IQR 56.5–75.0) and 55% were female. Most individuals were under treatment with TKIs (23/29, 79.3%) (CML On TKI) for a median of 10.7 years (IQR 3–13.5), while six participants (20.7%) had discontinued treatment (CML Off TKI) after a median of 3.5 years (IQR 2.5–6.3) after achieving a prolonged DMR. Nearly half (51.7%) of the individuals were on their first line of treatment. The two-dose vaccination schedule against COVID-19 was completed in all participants. The most frequently administered vaccine was Spikevax (16/29, 55.17%), followed by Comirnaty (9/29, 31%), and Vaxzevria (4/29, 13.8%).

Twenty healthy donors with a median age of 40 years (IQR 35–46) were included in this study as controls. Most healthy donors (60%) were female and all had received a complete two-dose vaccine of Comirnaty against SARS-CoV-2. Clinical and sociodemographic data of all participants are summarized in Table 1.

### 3.2. SARS-CoV-2 Breakthrough Infections

We monitored COVID-19 vaccine breakthrough cases during 17 months of follow-up assessments after receiving the full vaccination schedule. In the CML cohort, 34.4% (10/29) of individuals developed COVID-19 after being vaccinated. The median age of the participants with SARS-CoV-2 breakthrough infections was 63 years-old (IQR 57–73) and most were female (6/10; 60%). Four CML individuals (4/6; 67%) were on TFR and six CML individuals (6/23; 26%) were on treatment with TKI: nilotinib (3/23; 13%), imatinib (1/23; 4.3%), dasatinib (1/23; 4.3%), and asciminib (1/23; 4.3%). All infections with SARS-CoV-2 occurred after the second dose was administered and most individuals had been vaccinated with Spikevax (6/10; 60%), except three who were vaccinated with Comirnaty (3/10; 30%), and one who was vaccinated with Vaxzevria (1/10; 10%). All infections were mild and treated ambulatory (Table 2).

Eight healthy donors (8/20; 40%) reported SARS-CoV-2 breakthrough infection after receiving the second dose of Comirnaty. Most individuals were female (5/8; 62.5%) with a median age of 38 years-old (IQR 31–42) and without significant comorbidities. COVID-19 was mild in all cases.

### 3.3. Humoral Response after Two Doses of the Vaccine against SARS-CoV-2

**Figure 1:**
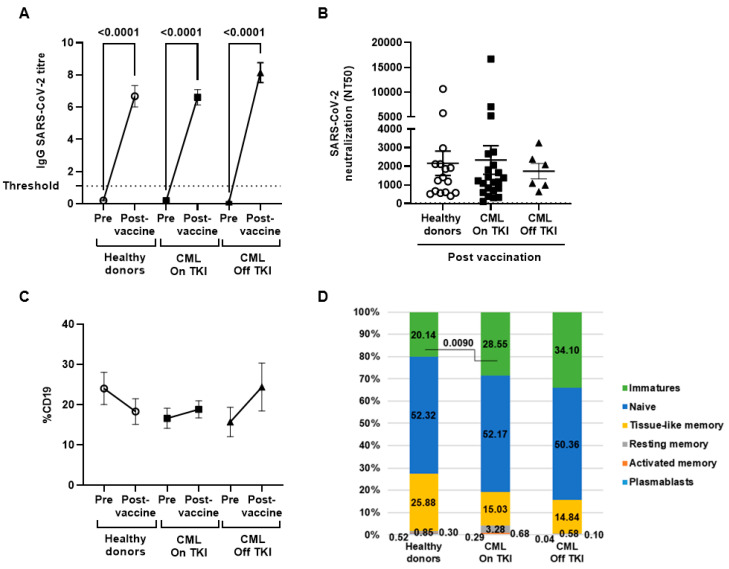
Humoral response against SARS-CoV-2 in individuals with CML before and one month after receiving the complete vaccination schedule against COVID-19. (**A**) Total IgGs levels against SARS-CoV-2 in plasma of the participants before and after complete vaccination. (**B**) Neutralizing capacity of specific IgGs in seropositive individuals after complete vaccination. (**C**) Total CD19+ B-cells in all the participants before and after vaccination. (**D**) B-cells subpopulations distribution in all the participants after complete vaccination. Each dot corresponds to the mean ± standard error of the mean (SEM). Each symbol represents a different cohort: healthy donors (open circles), CML On TKI (closed squares), and CML Off TKI (closed triangles). Wilcoxon signed-rank test was applied to calculate the statistical significance within groups. Kruskal—Wallis one-way and Mann—Whitney U of variance by rank were applied to calculate the statistical significance between groups.

Levels of IgG against SARS-CoV-2 were increased in all individuals with CML, similar to that of healthy donors (*p* < 0.0001) (Figure 1A). Neutralizing activity of IgGs was achieved in 85% of the healthy donors; 96% of the individuals with CML On TKI and 100% of individuals with CML Off TKI (Figure 1B). 

The total levels of B cells remained unchanged after vaccination and no differences were observed between the cohorts (Figure 1C). Both groups of individuals with CML showed increased levels of immature B-cells in comparison to healthy donors; this comparison was statistically significant for the CML On TKI (1.9-fold, *p* = 0.0090) (Figure 1D).

### 3.4. Cytotoxicity of PBMCs from Individuals with CML

**Figure 2:**
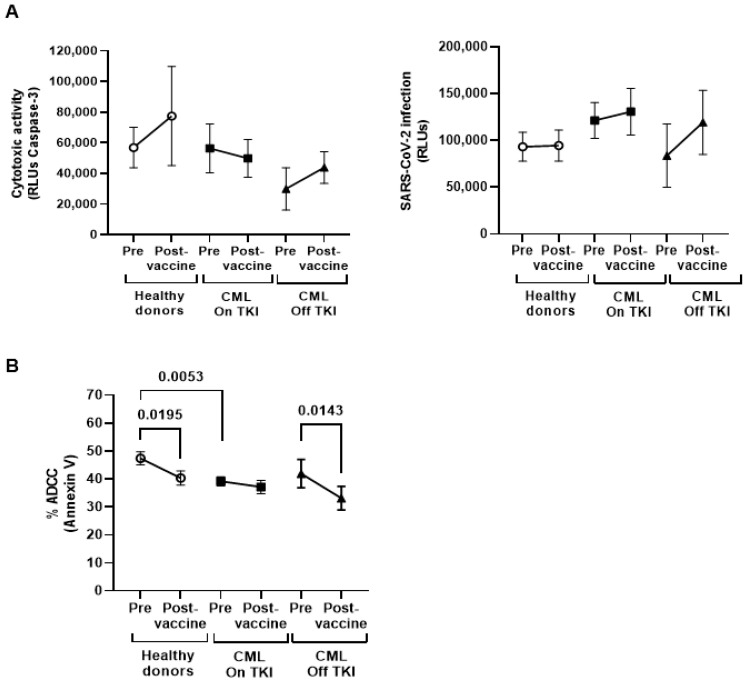
Cytotoxic activity against SARS-CoV-2 in individuals with CML before and one month after receiving the complete vaccination. (**A**) DCC was determined via chemiluminescence (RLUs) through measuring the activity of caspase-3 induced in a monolayer of Vero E6 cells infected with pseudotyped SARS-CoV-2 after co-culture with PBMCs isolated from the participants (left graph). The antiviral activity of PBMC against SARS-CoV-2-infected Vero E6 cells was evaluated via measuring the synthesis of Renilla by chemiluminescence (RLUs) (right graph). (**B**) The capacity of PBMCs to induce ADCC was determined via staining with Annexin V-PE rituximab-coated Raji cells after coculture with PBMCs. Each dot corresponds to the mean ± SEM. Each symbol represents a different cohort: healthy donors (open circles), CML On TKI (closed squares), and CML Off TKI (closed triangles). Paired *t*-test was applied to calculate the statistical significance within groups and ordinary one-way ANOVA was applied to calculate the statistical significance between groups.

We found no changes between groups in the capacity of PBMCs to induce DCC on pseudotyped SARS-CoV-2-infected Vero E6 cells (Figure 2A, left graph) or to interfere with viral replication (Figure 2A, right graph). ADCC activity was slightly reduced in PBMCs from individuals with CML On TKI in comparison to healthy donors (*p* = 0.0053) although no significant differences were found with the group of CML Off TKI (Figure 2B).

### 3.5. Phenotyping of Cytotoxic Cells Populations before and after Vaccination

**Figure 3:**
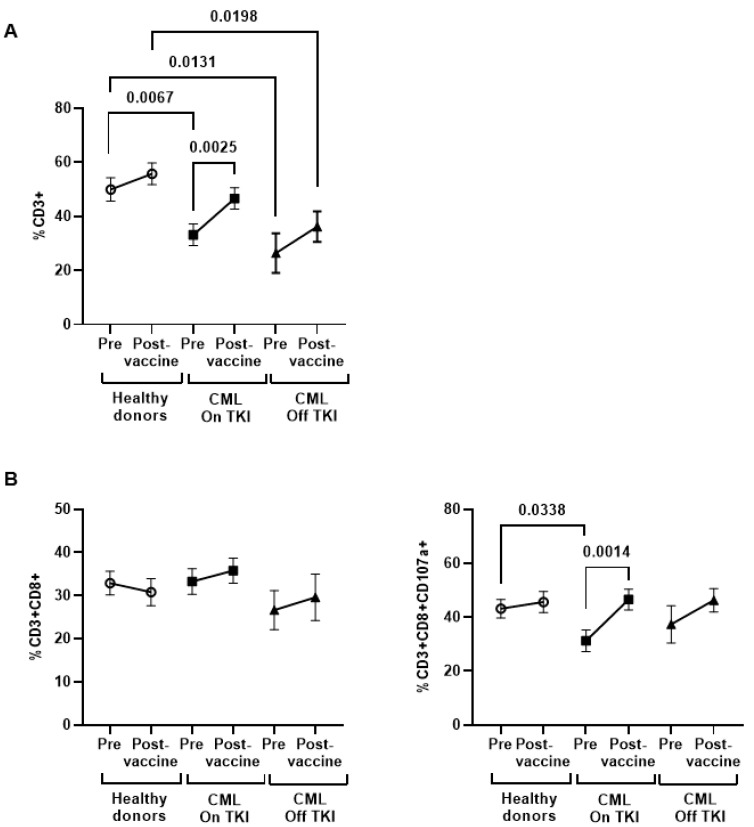
Levels of cytotoxic cells in PBMCs of individuals with CML before and one month after receiving the complete vaccination. (**A**) Analysis via flow cytometry of the levels of total CD3^+^ cells in PBMCs from the participants. Levels of CD8^+^ T cells (**B**) and expression of the degranulation marker CD107a in these cells in PBMCs from the participants. Each dot corresponds to the mean ± SEM. Each symbol represents a different cohort: healthy donors (open circles), CML On TKI (closed squares), and CML Off TKI (closed triangles). Paired *t*-test was applied to calculate the statistical significance within groups and ordinary one-way ANOVA was applied to calculate the statistical significance between groups.

**Figure 4:**
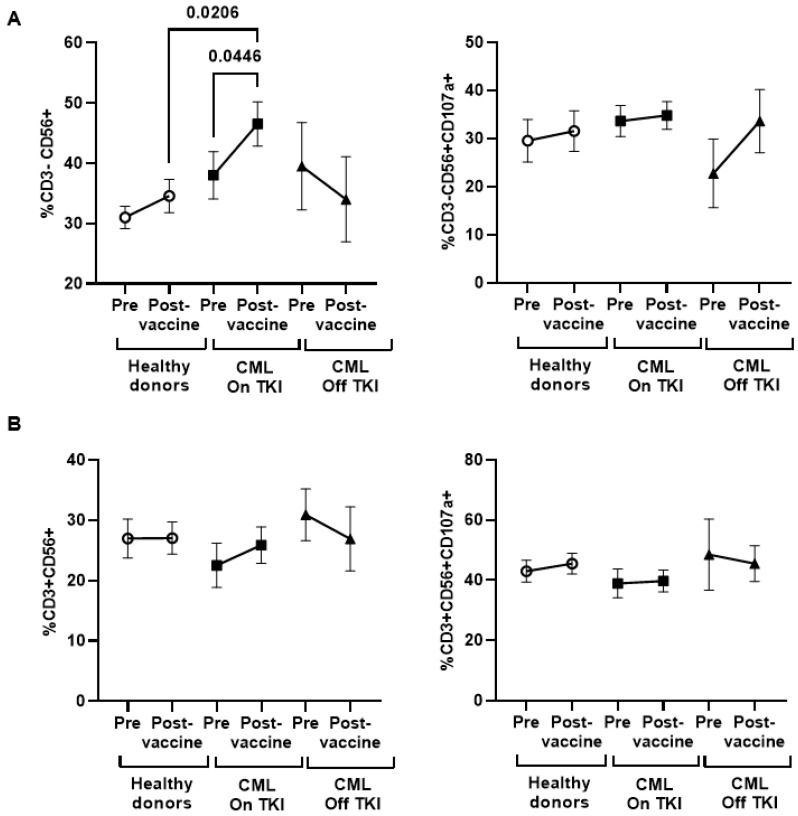
Analysis of NK and NKT-like cells in PBMCs of individuals with CML before and one month after receiving the complete vaccination. (**A**) Levels of NK cells (CD3^−^ CD56^+^) (left graph) and the expression of the degranulation marker CD107a in these cells (right graph) in PBMCs from the participants. (**B**) Levels of NKT-like cells (CD3^+^ CD56^+^) (left graph) and the expression of CD107a in these cells (right graph). Each dot corresponds to the mean ± SEM. Each symbol represents a different cohort: healthy donors (open circles), CML On TKI (closed squares), and CML Off TKI (closed triangles). Wilcoxon signed-rank test was applied to calculate the statistical significance within groups. Kruskal—Wallis one-way and Mann—Whitney U of variance by rank were applied to calculate the statistical significance between groups.

PBMCs from the supernatant of Vero E6 cells were analyzed via flow cytometry to determine the cytotoxic cell populations that were responsible for DCC activity. Total lymphocytes (CD3^+^) decreased 1.5-fold (*p* = 0.0067) in CML On TKI individuals and 1.9-fold (*p* = 0.0131) in CML Off TKI individuals before vaccination, in comparison to the healthy donors (Figure 3A). CD3 expression levels increased in both groups of CML participants after vaccination and this comparison was significant for CML Off TKI individuals in comparison to healthy donors (1.5-fold; *p* = 0.0198). No changes were observed in the levels of CD8^+^ T cells between groups (Figure 3B, left graph), although the expression of the degranulation marker CD107a reduced 1.4-fold (*p* = 0.0338) in CD8^+^ T cells from the CML On TKI individuals before vaccination (Figure 3B, right graph), although it increased 1.5-fold (*p* = 0.0014) after vaccination, in comparison to the healthy donors. The levels of NK cells were increased in CML individuals on treatment with TKIs in comparison to healthy donors, and this increase was significant after vaccination (1.3-fold, *p* = 0.0206) (Figure 4A). No significant changes were observed in the levels of NK (Figure 4B) or Tγδ cells between groups or in the expression of CD107a in these cells (Supplementary Figure S1).

## 4. Discussion

CML has a unique temporal course within the group of OHDs because of the direct efficacy of the treatment with TKIs over the leukemia, but also due to the sustained immunomodulatory anticancer effect of these drugs that permits treatment discontinuation in a selected group of individuals [22]. This latter effect has been attributed to the expansion of large granular lymphocytes (CD8^+^, NK and NKT) with a wide cytotoxic profile [9,23,24,25,26] and antitumoral effect, that have also been related to protection against some viral infections even after treatment discontinuation [27,28]. In fact, during the first waves of COVID-19 pandemic, before vaccination began, individuals with OHDs who were infected with SARS-CoV-2 developed severe forms of COVID-19 with a high incidence of critical and mortal illness [2,3,4], while only 5% of individuals with CML developed severe or critical COVID-19 during the same period [29].

Some studies have established that individuals with CML under TKI treatment may develop proper T-cell responses after vaccination, as is the case with the vaccine against influenza virus [13,30], and that these responses may be a consequence of the immunomodulatory influence of TKIs over cytotoxic populations [13,27]. High levels of antibodies against several vaccinated and non-vaccinated pathogens have also been described in individuals with CML [27,31]. However, B-cell impairment may occur as well due to the suppressive effect of some TKIs on the Bruton tyrosine kinase [13] that is essential for BCR signaling and B cell survival [32]. In fact, vaccination against pneumococci in CML individuals under TKI treatment was observed to be significantly impaired [13]. Consequently, the immune response developed in individuals with CML may not be the same for all pathogens and it may be different depending on if these individuals are on treatment with TKIs or during TFR.

Therefore, there was no certain knowledge about the efficacy of the immune response in CML individuals when vaccination against COVID-19 started in 2020. It was reported that seroconversion in individuals with CML was increased and similar to that of healthy donors after receiving one dose of vaccine against SARS-CoV-2 [33]. These antibodies showed a proper neutralizing activity and also described an adequate T-cell functionality based on cytokines measured after exposing cytotoxic cells to SARS-CoV-2 specific peptides [34]. Our study group also demonstrated that there was a direct cytotoxic response against target cells infected with pseudotyped SARS-CoV-2 in PBMCs from CML individuals and that the immune response reduced viral replication in these cells after receiving one single dose of the vaccine [31]. Later reports confirmed an increase in neutralization title against SARS-CoV-2 developed in individuals with CML after subsequent vaccine doses [33], although studies regarding the cellular response and the persistence of immunological memory are still scarce. 

In this study, we recruited a cohort of 29 individuals diagnosed with CML who received a complete vaccination schedule of two doses against COVID-19 and were then followed for 17 months. We confirmed the higher seroconversion rates and neutralizing capacity of antibodies developed in these individuals in response to vaccination, despite the high levels of immature B-cells. Regarding the cellular immune response, we observed similar direct cytotoxic capacity of the PBMCs isolated from individuals with CML to those obtained from healthy donors, despite the significant lymphopenia that was even higher in participants on TFR. Some impaired T-cell responses were found, such as the decreased degranulation capacity of CD8^+^ T cells from CML individuals on TKI before vaccination, although this activity was recovered after receiving the full vaccination schedule. ADCC response was also slightly reduced in individuals with CML on TKI and it significantly decreased in participants on TFR after vaccination. However, on the whole, the cellular immune responses developed in individuals with CML after vaccination was comparable to that of healthy donors.

When vaccination against COVID-19 began, there was a big concern regarding the loss of immunity over time in both individuals with OHDs and in healthy donors [35]. Moreover, the arrival of new variants of SARS-CoV-2 provided a new challenge to the efficacy of the immunity developed after vaccination due to the appearance of new peaks in the incidence of COVID-19 even among properly vaccinated individuals [17]. Our study showed similar results to those observed for immunocompetent individuals and demonstrated that complete vaccination against COVID-19, even with the administration of additional booster doses, does not prevent COVID-19 infection: it efficiently protects against severe and critical illness [36]. Nearly 35% of CML individuals had breakthrough infections of SARS-CoV-2 during the follow-up, similar to those of healthy donors (40%). All the infected participants had a mild form of COVID-19, which was a much lower rate than what was expected before vaccination. Interestingly, breakthrough infections were more frequent (67%) within the group of CML individuals who were on TFR after a median of 3.5 years, which indicated that the protective, immunomodulatory effect of TKIs appeared to be essential for the increase in the immune response developed by individuals with CML.

One limitation of this study was the reduced number of participants with CML on TFR who were recruited. However, this group of individuals that are less common in clinical settings should also be considered to evaluate the efficacy of COVID-19 vaccination.

## 5. Conclusions

In conclusion, the complete vaccination schedule against COVID-19 in CML individuals showed similar results in terms of humoral and cellular immunity to that of healthy donors, with slightly better responses in those individuals who were on treatment with TKIs in comparison to those who were on TFR, most likely due to the persistent immunomodulatory effect of these drugs. In addition, as with the healthy donors, vaccination did not impede breakthrough infections completely, although it did prevent the development of severe or critical illness in this special population of individuals with OHD. Although we obtained statistical significance in the comparison between groups, more studies are necessary to confirm these results in a large population.

## Figures and Tables

**Table 1:** Sociodemographic and clinical data of the individuals recruited for this study.

	CML On-TKI	CML Off-TKI	Healthy Donors
Participants	23	6	20
Age, median years (IQR)	65 (57–69)	76 (59–82)	40 (35–46)
Gender (female), n (%)	13 (56)	3 (50)	12 (60)
Time with CML, years (IQR)	9 (4–15)	15 (12.5–23.3)	-
Time on treatment with TKIs, years (IQR)	10.7 (3–13.5)	-	-
Time on TFR, median years (IQR)	-	3.5 (2.5–6.3)	-
First line treatment, n (%)	12 (52)	3 (50)	-
TKI, n (%)			
Ponatinib	3 (13)	-	-
Bosutinib	2 (9)	-	-
Nilotinib	6 (26)	-	-
Imatinib	6 (26)	-	-
Dasatinib	5 (22)	-	-
Asciminib	1 (4)	-	-
Vaccine, n (%)			
Spikevax	14 (61)	2 (33)	-
Comirnaty	5 (22)	4 (67)	20 (100)
Vaxzevria	4 (17)	-	-
SARS-CoV-2 breakthrough infections, n (%)	6 (26)	4 (67)	8 (40)

CML, Chronic myeloid leukemia; TFR, Treatment-free remission; TKI, Tyrosine kinase inhibitors.

**Table 2:** Sociodemographic and clinical data of COVID-19 vaccine breakthrough cases within the participants with CML who participated in the study.

Participant ID	Gender	Age (Years)	Vaccine	Current TKI	Time Since 2nd Dose to Infection (Months)
1	M	82	Comirnaty	-	13.5
2	F	75	Comirnaty	-	10.7
3	F	59	Spikevax	-	8.1
4	M	52	Comirnaty	-	1.5
5	F	69	Vaxzevria	Nilotinib	8.6
6	M	57	Spikevax	Nilotinib	7.4
7	F	68	Spikevax	Nilotinib	6
8	M	73	Spikevax	Asciminib	8.5
9	F	56	Spikevax	Imatinib	11.6
10	F	58	Spikevax	Dasatinib	7.1

CML, Chronic myeloid leukemia; F, Female; M, Male; TKI, Tyrosine kinase inhibitor.

## Data Availability

All data generated in this study have been included in this report.

## Supplementary Materials

The following supporting information can be downloaded at: https://www.mdpi.com/article/10.3390/cancers15205066/s1, Figure S1: Characterization of TCRγδ+ cells from CML individuals and healthy donors before and after receiving the vaccine.
