# Supplementary material for: Regular Humoral and Cellular Immune Responses in Individuals with Chronic Myeloid Leukemia Who Received a Full Vaccination Schedule against COVID-19
Source: Cancers (Basel). 2023 Oct 20;15(20):5066. doi: 10.3390/cancers15205066 (PMC10604981; doi:10.3390/cancers15205066)

Supplementary Figure 1. Characterization of TCRγδ+ cells from CML individuals and healthy donors before and after receiving the vaccine. Each dot corresponds to the mean ± standard error of the mean (SEM). Each symbol represented a different cohort: healthy donors (open circles), CML On TKI (closed squares), and CML Off TKI (closed triangles). Wilcoxon signed-rank test was applied to calculate the statistical significance within groups. Kruskal-Wallis one-way and Mann-Whitney U of variance by rank were applied to calculate the statistical significance between groups.

A

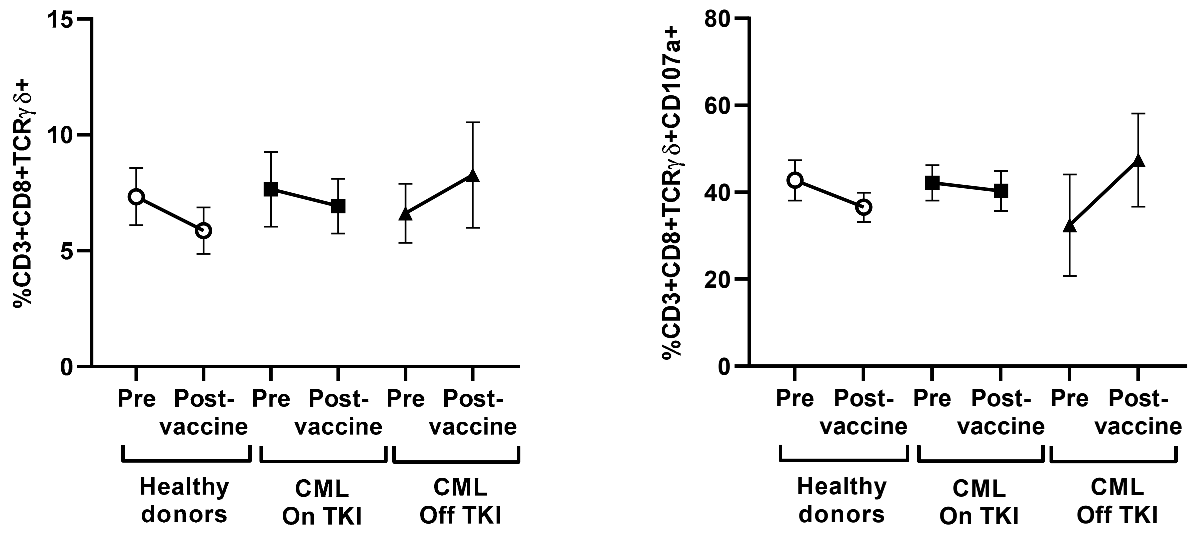

B

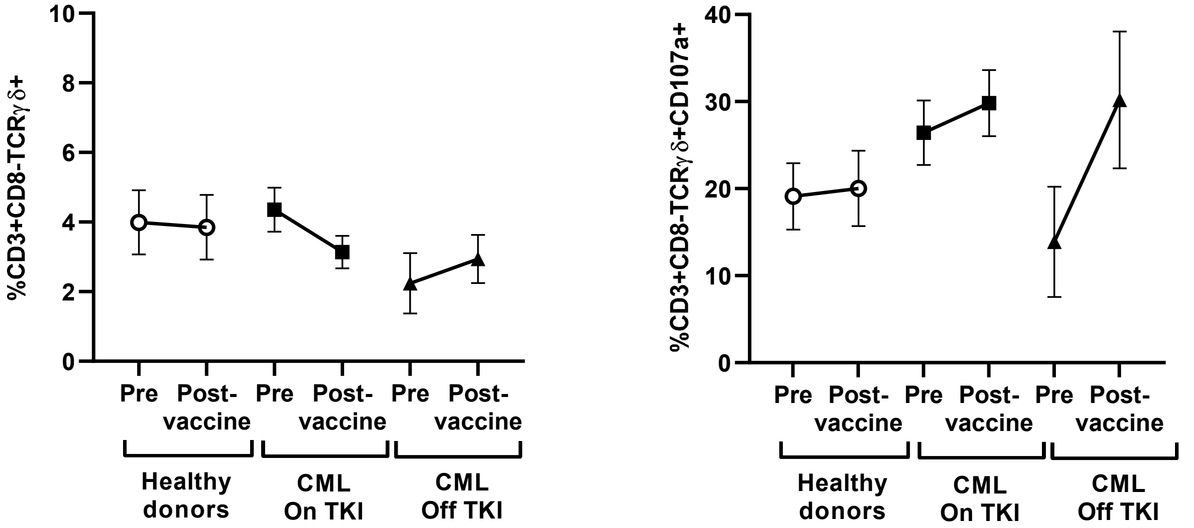

Supplement: Supplementary file 1 [file cancers-15-05066-s001.zip › cancers-2591744-SI.pdf]
